# New Addictions in Late Adolescence and Emerging Adulthood: How Attachment Style May Predict Problematic Use of Social Networks and Binge-Watching

**DOI:** 10.3390/healthcare12050556

**Published:** 2024-02-28

**Authors:** Renata Tambelli, Francesca Favieri, Maria Casagrande

**Affiliations:** Department of Dynamic and Clinical Psychology and Health Studies, “Sapienza” University of Rome, Via degli Apuli 1, 00185 Rome, Italy; renata.tambelli@uniroma1.it (R.T.); maria.casagrande@uniroma1.it (M.C.)

**Keywords:** attachment style, behavioral addiction, social network, binge watching

## Abstract

As behavioral addictions (BAs) significantly affect well-being, paying attention to the characteristics associated with their onset is relevant. Current studies suggest that BAs should be addressed from an adaptive–maladaptive continuum perspective to define what and how some behaviors became problematic. The multi-faceted nature of behaviors attributed to possible BA involves psychological and individual backgrounds (e.g., attachment style). Given its role in affecting personality processes, social development, and motivational drives, the attachment style would be involved in addiction-like behaviors from adolescence, defined as a critical period for BA onset. This study analyzed the association between the attachment dimensions and two possible BAs that can be included in an adaptive–maladaptive continuum (i.e., social network use and TV series watching). A sample consisting of 493 late adolescents/emerging adults (age range: 18–24) completed questionnaires assessing social network use, TV series watching, and attachment style. The results showed a positive association between problematic attachment styles and BAs. High worry and need for relationships (anxious attachment) would be risk factors for problematic social network use and relationships as secondary (avoidant attachment) would be a risk factor for problematic TV series watching. These findings suggest the importance of further analyzing the role of attachment styles and their dimensions in influencing behavioral expression early to prevent the occurrence of BA.

## 1. Introduction

In recent years, researchers have focused on the analysis of behavioral addictions (BAs; behaviors that, in their compulsivity and interference with daily functioning, follow similar features of drug addictions). The first obstacle to overcome is the categorization of potentially maladaptive behaviors that could become BA. Indeed, as suggested by Billieux [1], the risk of overpathologization of common behaviors may be ascribed to the atheoretical and confirmatory approach of BA studies [2]. This is even more true in the context of the “new addictions” that involve technologies, devices, and habits that are cross-sectionally adopted by populations and can be characterized by compulsivity or lack of control (e.g., gaming, internet, social media, and smartphones) [3,4] but that are widely used in modern society, making it difficult to distinguish their positive and negative features.

For these reasons, the concept of a range of behaviors on a continuum from leisure to problematic activity [1] is much more sustainable than the concept of a pathological categorization of specific behaviors in BA. Accordingly, instead of focusing on the behavior itself, researchers should focus on the characteristics and risk factors that may influence the behavioral trajectory into this continuum. For example, studies on the internet, gaming, or TV series showed that, on the one hand, they are associated with positive outcomes and psychological well-being (e.g., low anxiety and distress, high mood, and low impulsivity), defining them as leisure activities, but, on the other hand, they are associated with negative psychological outcomes (such as high anxiety, a sense of loneliness, and depression) in their problematic expression [5,6,7]. This apparent divergence can be ascribed to various reasons, such as time spent enacting these behaviors, device accessibility, personological traits, and social and environmental contexts [1,5,6].

Regardless of the perspective, it appears clear that many suggested BAs are multi-faceted and characterized by the heterogeneity of expression and related factors, including psychopathological dimensions, personological traits, and aspects ascribable to the individual’s background. Moreover, it is clear that it is important to detect the features of each behavior to understand if and to what degree its expression from a leisure activity becomes problematic (i.e., characterized by an increased risk of occurrence of BA). In this sense, the endogenous facets may influence the exogenous expression of the behavior that could decline into BAs. For example, if we refer to social network use (SN), defined as access to web social pages (such as Instagram, Facebook, Tik-Tok, Twitter, etc.) and involving interaction and communication with other users by posting information, comments, messages, images, etc. [8], it is characterized by the tendency to interact with others in a virtual rather than real world, either actively or passively [9]. In contrast, another possible BA, referred to as binge-watching (BW), is characterized by the concept of multiple episodes of TV content in a single session, usually alone, with reduced social interactions during the experience [10]. Regardless of their features on social interaction, both behaviors affect mental health positively and negatively to different degrees [11,12]. However, from these preliminary definitions, it seems evident that characteristics of interpersonal interactions could differently influence the continuum of each behavior.

Among the aspects that can influence the pathologization of a common behavior are certainly the mental representations and the motivations that drive it [13,14,15]. In this sense, one of the aspects that should be deepened in at-risk behavior is the attachment style [16,17,18,19]. The attachment theory [20,21] was conceptualized to describe the characteristics behind the bonding between human beings and focused on the framework of interpersonal influence, starting from infant–caregiver interactions to adults’ close relationships. The attachment theory has been employed to analyze personality processes, social development, and motivational drives, and studies have reported the involvement of attachment styles in substance and behavioral addiction [13,22,23]. In fact, according to clinicians, the attachment style and its features are associated with addiction, which may be ascribed to an attachment disorder resulting from the achievement of relational independence from the caregiver and expressed as an attempt—especially in late adolescence or young adulthood—to manage their attachment strategies [17,24,25]. Healthy attachment, characterized by positive and secure relationships and confidence toward others, helps individuals manage stress and environmental demands. In contrast, problematic attachment styles (from insecure to disorganized) generate unhealthy coping toward problems and are associated with a worse psychological status. This last case includes risky behaviors such as BA or problematic approaches to common activities that may decline in a diagnosis of BA [26]. In this sense, it is relevant for researchers and clinicians to understand what and how attachment characteristics affect behaviors and their adaptive–maladaptive expression. It will allow the definition of possible risk factors for behavioral addictions or protective dimensions on which skills are built and empowered to reduce the risk of problematic behaviors before their pathological drift. Previous studies have examined the relationship between attachment style and SN addiction (for a review, see Stoven and Herberg, 2020 [27]), reporting heterogeneous results due to the lack of consistency in the assessment and definition of the attachment dimensions. However, these studies generally suggested an association between insecure attachment and increased risk of SN, with adolescents’ attachment to parents and friends [14] playing a role in influencing behavioral manifestations both directly and indirectly via psychopathological conditions. Instead, to our knowledge, no studies have analyzed the association between BW and attachment style, both considering the general population and specific target populations such as adolescents at different stages and young adults.

According to these premises, this study aimed to establish the association between the dimensions (i.e., Discomfort with Closeness, Need for Approval, Preoccupation with Relationships, and Relationships as Secondary) [28], which determine different attachment styles [20] and the adaptive–maladaptive continuum of social network use and TV series watching, verifying whether different behaviors in terms of expression and social interaction are differentially associated with attachment characteristics. We hypothesized that problematic features of social network use in a sample of late adolescents and emerging adults might be affected by alteration in the attachment dimensions rather than problematic binge-watching. Moreover, we expected that worrying about relationships, as an expression of an anxious attachment style, would be significantly associated with social network use, whereas the tendency to distance oneself from others as an expression of an avoidant attachment style would be significantly associated with binge-watching behavior.

## 2. Materials and Methods

### 2.1. Procedure and Ethical Consideration

A cross-sectional survey disseminated online via the main social network of the Health Psychology Laboratory (“Sapienza” University of Rome) between March and May 2023 collected data from Italian late adolescents. The Ethical Committee of the Department of Dynamic, Clinical Psychology and Health Studies of “Sapienza” University of Rome (protocol code 0000683 and date of approval 2 May 2022) approved the study procedure, and each respondent confirmed their acceptance to participate in the study by signing informed consent before the questionnaire section. The informed consent presented the main objective and procedure of the survey, which required approximately 30–40 min to complete.

### 2.2. Participants

Considering the aim of the study, to define the minimum sample size, the formula for cross-sectional studies on qualitative variables suggested by Charan and Bisawas [29] was adopted $n=\frac{Z^{2}\times p\left(1-p\right)}{d}$. Considering that the prevalence of some digital addiction was reported in 25 percent of the general population [30], this study should include at least 288 participants. At the end of the survey dissemination time, 493 late adolescents and emerging adults (age range: 18–24; 70% females) completed the questionnaires and were included in the analyses. Table 1 shows the main characteristics of the sample.

### 2.3. Instruments

Demographic characteristics and habits: the first items allowed us to collect general information about the respondents (e.g., age, sex, education, and habits related to internet and Social Network use and TV series watching).

The Brief screening for Social Network Addiction Risk (BSNA; Italian version [30]) was used to assess social network use. The scale was composed of eleven items on a 5-point Likert scale (0 = never; 4 = always) assessing behavioral modification (subscale 1) and motivation (subscale 2) regarding the use of social networks. The global score (the sum of the two subscales) provides a global risk for social network addiction. The scale showed high reliability (Cronbach’s α: Factor 1 = 0.84, Factor 2 = 0.82, Total Score = 0.87). Higher scores on this screening scale indicated a high risk of problematic SN, defined as an expression of the risk of occurrence of BA to detect further [31].

The Binge-Watching Addiction Questionnaire (BWAQ; Italian version [32]) was adopted to assess the behavior related to watching TV series. The scale includes 20 items scored on a 5-point Likert scale (0 = never to 4 = always) assessing the risk of compulsive BW with four subscales (Carving, Avoidance, Dependency, and Tolerance) and a global score. Specifically, total BWAQ scores higher than 51 are defined as a cut-off to define moderately problematic and highly problematic behaviors characterized by traits reported in BA for further analysis. The questionnaire is reliable for both the subscales and the global score (Cronbach’s α: Factor 1 = 0.91, Factor 2 = 0.82, Factor 3 = 0.75, Factor 4 = 0.81, Global Score = 0.84) [32].

The Attachment Style Questionnaire (ASQ; Italian version [28]) was used to measure five dimensions of attachment style with forty items rated on a 6-point scale (from 1 = totally disagree to 6 = totally agree). The features of the attachment style were Confidence, Discomfort with Closeness, Need for Approval, Preoccupation with Relationships, and Relationships as Secondary. The five scales are associated with two latent factors as expressions of attachment style, i.e., Anxiety and Avoidance [18]. Adequate reliability was reported for each questionnaire scale (Cronbach’s α: Confidence = 0.70, Discomfort with Closeness = 0.71, Relationship as Secondary = 0.67, Need for Approval = 0.74, Preoccupation with Relationships = 0.71) [28].

### 2.4. Data Analysis

Statistical analyses were performed using the free software Jamovi (Version 2.3) [33].

The first descriptive analysis allowed us to detect the distribution of categorical variables and means and standard deviations of continuous variables. Non-parametric statistics (the Kruskal–Wallis test and Wilcoxon–Mann–Whitney test) were conducted considering habits related to SN and TV-series watching as grouping variables and the global scores of BSNA and BWAQ as dependent variables.

The prevalence of problematic behavior on the internet and watching TV series was also calculated considering the cut-off of BWAQ (>51) and BSNA (>36) scales for each participant and categorizing them as leisure activities if the scores were lower or equal to cut-offs and “problematic” if the scores were higher than cut-offs. Considering this categorization, χ^2^ analyses were conducted to test if the information related to demographics and habits could be associated with the different expressions of the behaviors.

To test the association between continuous variables assessed by the standardized questionnaires, the normality of the data was tested using the Shapiro–Wilk test, which reported a non-normal distribution for all the variables (all *p* < 0.001). For this reason, non-parametric Spearman’s correlations were conducted between the variables to explore the relationship between the ASQ scales and the behaviors assessed by the BWAQ and BSNA, including their subscales.

Subsequently, to test the hypotheses of the study, two multiple regression analyses were independently carried out for binge-watching behavior and social network behaviors, considering the dimensions of attachment assessed by the ASQ (Confidence, Discomfort with Closeness, Need for Approval, Preoccupation with Relationships, and Relationships as Secondary) as predictors. 

Finally, according to ASQ scores, the risk ratio for problematic BW and SN was calculated using binomial logistic regression, including the categorization of BW as leisure and problematic behaviors according to the BWAQ [32] and BSNA [31] cut-off scores as dependent variables.

## 3. Results

### 3.1. Descriptive Data

Table 2 shows the means and standard deviations of the participants’ results on the questionnaires and the distribution of participants according to the cut-off for SN and BW.

No significant differences in the proportions of problematic SN (χ^2^ = 4.33; *p* = 0.11) and BW (χ^2^ = 5.07; *p* = 0.08) were reported considering relationship status, as well as considering education (SN: χ^2^ = 0.69; *p* = 0.71; BW: χ^2^ = 0.27; *p* = 0.87).

The Kruskal–Wallis test, considering the category of time spent on social networks, reported significant differences in scores of BSNA (χ^2^ = 74.0; *p* < 0.001) and BWAQ (χ^2^ = 21.8; *p* < 0.001). Specifically, higher scores on the BSNA were reported in those who spent more than 8 h a day on SN compared to the other categories (all *p* < 0.01). Moreover, spending 1–2 h daily on SN is characterized by lower SN scores on the BSNA compared with the other categories (all *p* < 0.03). Considering BWAQ scores, significant differences were reported between those who spent less than 1 h a day and those who spent 3–5 h (W = 4.38; *p* = 0.02) and 6–7 h (W = 5.74; *p* < 0.001) as well between those who spent 1–2 h compared to those who spent 6–8 h (W = 4.69; *p* = 0.01). Less time spent on SN included lower scores in BWAQ.

The Kruskal–Wallis test considering the type of user did not report significant differences between the categories in BSNA scores (χ^2^ = 0.08; *p* = 0.78) or BWAQ scores (χ^2^ = 3.60; *p* = 0.07).

The Wilcoxon–Mann–Whitney test considering classification for dating app. reported that those who did not belong to a dating app had lower scores on the BSNA (U = 2.90; *p* = 0.002) but did not differ significantly in BWAQ scores (U = 0.54; *p* = 0.30). Similarly, no significant differences emerge considering the variable TV series watching (U = 0.32; *p* = 0.62).

### 3.2. Attachment Dimensions Associated with BW Behavior

Spearman’s correlations between ASQ and BWAQ indices revealed significant associations between the variables. Specifically, the total score of the BWAQ significantly increased with increasing levels of Discomfort with Closeness (*ρ*_s_ = 0.13; *p* = 0.005), Need for Approval (*ρ*_s_ = 0.19; *p* < 0.001), Preoccupation with Relationships (*ρ*_s_ = 0.22; *p* < 0.001), and Relationships as Secondary (*ρ*_s_ = 0.10; *p* = 0.04). Moreover, BWAQ subscales were associated with ASQ variables (see Table 3).

A multiple linear regression considered the total score of BWAQ as the dependent variable and ASQ scales as predictors. The model resulted in being significant despite the very low variance explained (R^2^_adjusted_ = 0.05; F_5,487_ = 6.12; *p* < 0.001; see Table 4), with high levels of preoccupation with relationship predicting higher BWAQ scores.

According to the classification of BW in leisure (a score lower or equal to 51) or problematic (a score higher than 51), logistic binomial regression was carried out, considering the ASQ scales as predictors, and the results reported that high scores in Relationship as secondary are 1.12 times more likely in those who experienced problematic BW than in those who adopted BW as a leisure experience (OR = 1.12 [1.01–1.24]; z = 2.22; *p* = 0.03).

### 3.3. Attachment Dimensions Associated with SN Behavior

Pearson’s linear correlation between ASQ and BSNA indices reported that total BSNA was negatively correlated with Confidence levels (*ρ*_s_ = −0.8; *p* < 0.001) and positively correlated with Discomfort with Closeness (*ρ*_s_ = 0.21; *p* < 0.001), Need for Approval (*ρ*_s_ = 0.40; *p* < 0.001), Preoccupation with Relationships (*ρ*_s_ = 0.40; *p* < 0.001), and Relationships as Secondary (*ρ*_s_ = 0.12; *p* = 0.007). Also, BSNA subscales are significantly associated with BSNA (Table 5).

A multiple linear regression considered the total score of BSNA as the dependent variable and ASQ scales as predictors. The model resulted in being significant (R^2^_adjusted_ = 0.21; F_5,487_ = 26.7; *p* < 0.001), with high levels of discomfort for closeness and need for approval predicting higher BSNA scores (see Table 6).

According to the classification of SN as a leisure activity (a score lower or equal to 36) or problematic and at-risk behavior (a score higher than 36), a logistic binomial regression was carried out considering the ASQ scales as predictors. The results confirmed that high scores in Need for approval are 0.97 (OR = 0.97 [0.93–0.99]; z = 2.35; *p* = 0.02) and high scores in Preoccupation with relationship are 0.94 (OR = 0.94 [0.91–0.99]; *p* < 0.001) times more likely in those who experienced problematic SN use than in those who experienced SN as a leisure activity.

## 4. Discussion

Previous literature indicates the role of attachment styles in the onset and exacerbation of psychopathologies [34,35], as well as the onset of problematic behaviors [36,37,38,39]. It is well known that the early attachment experience affects adult relationship styles [40]. Accordingly, both early attachment experiences (i.e., the child–caregiver relationship) and consequent adult attachment styles are associated with psychological or behavioral outcomes. Specifically, anxious and avoidant dimensions are particularly related to health risk behaviors [36]. However, when we referred in this study to attachment style, the perspective was a dimensional approach suggested as more valid in detecting attachment theory characteristics [28]. From this perspective, the way to interact with others is a consequence of early childhood experiences but is not strictly framed in rigid categories. Keeping in mind these aspects, the main aim of this study was to clarify the relationship between some dimensions that characterized adult attachment style and the onset of behaviors that may be included in an adaptive–maladaptive continuum affecting the quality of life and well-being. Particularly, this relationship was detected in a sample including late adolescents and emerging adults due to their reported high involvement in at-risk behaviors as well as in the onset of BA [6,41]. We decided to focus on two behaviors that have been indicated as possible “new addictions” characterized by different degrees of social interaction and included in a range from leisure activity—defining a positive psychological and social frame—to problematic expression—with similarities with addiction. These behaviors were SN use and BW [1].

In line with our hypotheses, the results of this study indicated interesting insights that could increase knowledge on the relationship between risk behaviors and attachment styles: (a) a positive association between problematic attachment styles (expressed by the dimensions of the ASQ) and problematic expression of both behaviors; (b) a predictive role of high worry and need for relationships (expression of anxious attachment, characterized by high anxiety levels) for problematic SN use; and (c) a predictive role of high relationship as secondary (expression of an avoidant attachment style) for problematic BW.

The first result found in this study confirms a certain grade of complexity of the BA. Previously, Manohar et al. [41], in an interesting synthesis of qualitative studies on BA, reported that there are multiple dimensions to consider when referring to problematic behaviors, especially in adolescence (age range: 10–20) when the onset is more likely. The authors highlighted the importance of referring to a “spectrum of BA”, defined as a continuum of severity (adaptive–maladaptive), and the impact on the quality of life of the behaviors that may evolve in addiction. In this sense, the authors’ perspective agrees with our view of focusing on vulnerability factors for the risk of engaging in BA.

To better understand our findings, it seems relevant to consider the specificities of each behavior to justify how attachment dimensions may affect behavioral expression. Cao et al. [42] explored the mechanism behind social media addiction and suggested that motivational factors, including perceived enjoyment and social interaction, play a role in increasing the use and implementing the risk of addiction in a vulnerability framework. Our results extend this risk framework by suggesting that it would be characterized by relational fragility: the perceived need to establish relationships and interact with others and the preoccupation with these relationships may lead late adolescents to prefer virtual rather than real interaction, increasing the risk of SN addiction, as previously suggested in the field of internet addiction. In this sense, attachment style may drive SN use in conjunction with other dispositional factors, such as interpersonal problems, personality, and emotion regulation [35,43,44]. This perspective was previously suggested by authors such as Liu and Ma [36] who reported an association between anxiety in experiences in close relationships as an attachment style and social media addiction, with the role of emotional dysregulation as a possible mediator. Moreover, a recent study by Marzilli and colleagues [45] on a sample of young Italian college students reported a dynamic relationship between attachment, alexithymia, and psychopathological risk in predicting internet addiction, also involving social networks.

Accordingly, further studies should continue to explore the nature of the relationship, considering other variables and verifying what factors in the adaptive–maladaptive continuum of SN use may affect the exacerbation of the behavior in younger people. As suggested by other authors [46], it would help prevent the impact of the social era on people and use the virtual world to enhance real relationships and not reduce or destroy them.

New evidence emerged from the findings on BW and its association with attachment dimensions assessed by the ASQ. BW is a recently defined phenomenon, and many studies have shown its dual nature: on the one hand, it can be useful for psychological well-being and can stimulate creativity and social interactions; on the other, BW is an at-risk behavior that may negatively affect mood and increase isolation and loneliness [32]. Our study is the first to link problematic BW to the avoidant attachment style. In particular, the risk of occurrence of problematic BW increases with an increased tendency to perceive relationships as secondary [26]. This result would be consistent with the results of studies indicating that the problematic typical pattern of BW is characterized by poor social interactions, preferring to dedicate a large amount of time to TV series in loneliness.

In conclusion, our evidence provides an interesting picture of at-risk behaviors among adolescents and the role of the attachment style in their expression. Furthermore, the results indicated that (i) while an anxious attachment style (with high anxiety and worrying about relationships) affects motivation and has interpersonal implications that can affect an activity directed toward social interactions (also if in a digital world), (ii) an avoidant attachment style would predict the negative features of BW.

Surely, some limitations of the study should be considered. One of these limitations is the lack of analyses of possible psychological dimensions involved in behavioral expression (such as emotional regulation, alexithymia, personological characteristics, or coping strategies) that may be associated with a bidirectional relationship with attachment styles and that previous studies included in models of interaction for behavioral addiction, such as pathological gambling, internet, or Facebook addiction [44,45]. Also, other lifestyles and demographic variables that may affect behavioral expression directly and indirectly should be considered. In fact, although demographics (education and relationship status) and habits related to SN and BW (time spent on social network platforms, types of users, and TV-series watching) were controlled in this study to detect if they affect the behavioral expression, further studies could consider all these variables in mediation or moderation models to define possible theories of the risk of BA, also involving multiple behaviors. It would also be interesting to further consider other characteristics of the users. For example, particularly for SN, it would be interesting to understand if SN interactions may be ascribed to work-related reasons or specifically associated with the urge to interact with others in a virtual world, as we hypothesized in this work. Another limitation is the low prevalence of problematic SN and BW. The small number of individuals who overcame the cut-off for problematic behavior expression in the scale could have affected the effect size of the results, reducing their generalizability and not allowing us to consider the impact of co-occurrence of the phenomenon and possible different patterns of attachment. It would also be interesting to analyze other characteristics of the phenomena, starting with the information related to the users’ styles, which we only included in descriptive statistics. The use of the ASQ allowed us to collect data on the dimensions involved in the expression of attachment styles; however, its use reduces the possibility of further inference about attachment styles. In fact, as pointed out by Fossati and colleagues [28], there are no mutually exclusive types of attachment in adults. Finally, although consistent with the aim of detecting widespread behaviors in a sample identified as “at-risk” for BA, further studies should include samples of early adolescents and adults to test for similarities and differences across the lifespan in the relationship between attachment and behavior.

## 5. Conclusions

Since BAs are increasing in terms of prevalence and impact on mental health, especially in young populations [46], detecting aspects that may be involved in the occurrence of problematic behaviors is a starting point for developing interventions for the prevention and care of overt disorders. As reported by previous studies, adolescence and emerging adulthood are critical periods for the occurrence of behavioral addictions [47] and are characterized by the stabilization of attachment styles, self-determination, and personological traits [48]. Our results underline a predictive role of attachment style and its dimensions in the negative expression of the behaviors included in an adaptive–maladaptive continuum that future studies should attempt to verify and generalize to other at-risk behaviors. Including this approach in studies on BA in the young population would successfully prevent the consolidation of maladaptive behavior from early manifestations. Moreover, if future studies deepen the multiple factors involved in the relationship, interventions can be directed toward coping strategies aimed at managing both maladaptive attachment styles and psychological dimensions influencing behavioral outcomes. Finally, the most relevant findings of this study concern the risk of both behaviors in adolescents and emerging adults. It is crucial to consider this risk according to their specific trajectories. Personal characteristics associated with attachment styles, such as the need for relationships, the tendency to isolate oneself, or considering social interactions as irrelevant, affect the risk of the occurrence of BA and the quality of life. The complexity of the interaction with psychological factors and psychopathological conditions (e.g., depression, anxiety, etc.) appears clear, as well as the need to consider it in both research and clinical fields.

## Figures and Tables

**Table 1:** Characteristics of the sample.

Variables	Total (n = 493)
Females (%)	345 (70.0)
Mean age (Std.dev)	22.2 (1.42)
Education (%)	
High School degree	247 (50.0)
Bachelor’s degree	221 (45.0)
Master’s degree	25 (5.0)
Relationship Status (n, %)	
Married	8 (1.7)
In a relationship	253 (51.3)
Single	232 (47.0)
Time spent on social network platforms * (n, %)	
No time spent on social networks	2 (0.2)
1–2 h per day	175 (35.5)
3–5 h per day	236 (47.9)
6–8 h per day	47 (9.5)
More than 8 h per day	33 (6.9)
User Type of Social Networks * (n, %)	
Active (active interaction with other users; bloggers; content sharing)	130 (26.3)
Passive (no active interaction with other users; scrolling of homepage with no reason)	361 (73.0)
No Users	2 (0.7)
Dating app (n, %)	
Yes	137 (27.7)
No	356 (72.3)
TV-series watching (n, %)	
Yes	475 (96.3)
No	18 (3.7)

* Social Network: i.e., Facebook, Instagram, Twitter (X), TikTok, Snapchat.

**Table 2:** Descriptive data of the questionnaire results and classification of participants according to BSNA and BWAQ global scores.

Variables	Mean (Standard Deviation)	CI 95%
BSNA		
Behavioral Modification	20.20 (6.69)	[19.6–20.8]
Motivation	12.30 (4.86)	[11.9–12.7]
Total score	32.50 (10.50)	[31.6–33.5]
BWAQ		
Craving	9.10 (6.82)	[8.50–9.71]
Avoidance	7.45 (3.91)	[7.11–7.80]
Dependency	3.84 (2.42)	[3.63–4.06]
Tolerance	1.35 (2.40)	[1.14–1.57]
Total Score	21.8 (12.8)	[20.6–22.9]
ASQ		
Confidence	31.10 (7.64)	[30.4–31.7]
Discomfort with Closeness	36.70 (8.57)	[35.9–37.5]
Need for Approval	13.6 (5.60)	[13.1–14.1]
Preoccupation with Relationships	23.9 (8.05)	[23.2–24.7]
Relationships as Secondary	28.2 (7.46)	[27.5–27.8]
Problematic SN	167 (33.9%)	
Problematic BW	12 (2.4%)	
Co-occurrence of problematic SN and Problematic BW	9 (1.8%)	

**Table 3:** Pearson’s linear correlation between ASQ and BWAQ indices.

	Total BWAQ	Craving	Dependency	Anticipation	Avoidance
Confidence	−0.03	−0.06	0.01	0.03	−0.03
Discomfort with Closeness	0.13 *	0.13 **	0.06	0.04	0.06
Need for Approval	0.19 *	0.19 ***	0.15 ***	0.06	0.14 **
Preoccupation with Relationships	0.22 ***	0.20 ***	0.22 ***	0.12 **	0.13 **
Relationships as Secondary	0.10 *	0.13 **	−0.02	0.008	0.15 **

* *p* < 0.05; ** *p* < 0.01; *** *p* < 0.001.

**Table 4:** Multiple linear regression analysis predicting BWAQ score.

	Β	SE	t	CI (95%)	*p*
Confidence	0.10	0.09	1.13	[−0.04–0.16]	0.26
Discomfort with Closeness	0.07	0.08	0.87	[−0.06–0.15]	0.38
Need for Approval	0.10	0.10	0.97	[−0.06–0.18]	0.33
Preoccupation with Relationships	0.31	0.10	3.26	[0.07–0.29]	<0.001
Relationships as Secondary	0.11	0.11	1.04	[−0.04–0.14]	0.30

**Table 5:** Spearman’s correlation (*ρ*_s_) between ASQ and BSNA indices.

	Total BSNA	Behavioral Modification	Motivation
Confidence	−0.18 ***	−0.10 *	−0.23 ***
Discomfort with Closeness	0.21 ***	0.18 ***	0.21 ***
Need for Approval	0.40 ***	0.35 ***	0.39 ***
Preoccupation with Relationships	0.39 ***	0.37 ***	0.36 ***
Relationships as Secondary	0.12 **	0.10	0.15 **

* *p* < 0.05; ** *p* < 0.01; *** *p* < 0.001.

**Table 6:** Multiple linear regression analysis predicting BSNA score.

	Β	SE	t	CI (95%)	*p*
Confidence	0.001	0.07	0.03	[−0.09–0.09]	0.98
Discomfort with Closeness	0.07	0.06	1.20	[−0.04–0.15]	0.23
Need for Approval	0.33	0.07	4.46	[0.14–0.36]	<0.001
Preoccupation with Relationships	0.34	0.07	4.85	[0.15–0.34]	<0.001
Relationships as Secondary	0.006	0.08	0.07	[−0.08–0.09]	0.94

## Data Availability

Data are available upon request to the corresponding authors.
